# Phosphorylation of a Myosin Motor by TgCDPK3 Facilitates Rapid Initiation of Motility during *Toxoplasma gondii* egress

**DOI:** 10.1371/journal.ppat.1005268

**Published:** 2015-11-06

**Authors:** Rajshekhar Y. Gaji, Derrick E. Johnson, Moritz Treeck, Mu Wang, Andy Hudmon, Gustavo Arrizabalaga

**Affiliations:** 1 Department of Pharmacology and Toxicology, Indiana University School of Medicine, Indianapolis, Indiana, United States of America; 2 Department of Biochemistry and Molecular Biology, Indiana University School of Medicine, Indianapolis, Indiana, United States of America; 3 Stark Neuroscience Research Center, Indiana University School of Medicine, Indianapolis, Indiana, United States of America; 4 The Francis Crick Institute, London, United Kingdom; MRC National Institute for Medical Research, UNITED KINGDOM

## Abstract

Members of the family of calcium dependent protein kinases (CDPK’s) are abundant in certain pathogenic parasites and absent in mammalian cells making them strong drug target candidates. In the obligate intracellular parasite *Toxoplasma gondii* TgCDPK3 is important for calcium dependent egress from the host cell. Nonetheless, the specific substrate through which TgCDPK3 exerts its function during egress remains unknown. To close this knowledge gap we applied the proximity-based protein interaction trap BioID and identified 13 proteins that are either near neighbors or direct interactors of TgCDPK3. Among these was Myosin A (TgMyoA), the unconventional motor protein greatly responsible for driving the gliding motility of this parasite, and whose phosphorylation at serine 21 by an unknown kinase was previously shown to be important for motility and egress. Through a non-biased peptide array approach we determined that TgCDPK3 can specifically phosphorylate serines 21 and 743 of TgMyoA *in vitro*. Complementation of the *TgmyoA* null mutant, which exhibits a delay in egress, with TgMyoA in which either S21 or S743 is mutated to alanine failed to rescue the egress defect. Similarly, phosphomimetic mutations in the motor protein overcome the need for TgCDPK3. Moreover, extracellular *Tgcdpk3* mutant parasites have motility defects that are complemented by expression of S21+S743 phosphomimetic of TgMyoA. Thus, our studies establish that phosphorylation of TgMyoA by TgCDPK3 is responsible for initiation of motility and parasite egress from the host-cell and provides mechanistic insight into how this unique kinase regulates the lytic cycle of *Toxoplasma gondii*.

## Author Summary

*Toxoplasma gondii* can cause severe disease and death in the immunocompromised and in those infected congenitally. Due to limitations of existing drugs there is a need for studying proteins that are unique and essential to the parasite. We recently established that TgCDPK3, a member of a family of calcium dependent protein kinase present in plants and some parasites but absent in human cells, regulates parasite egress from the host cell. While it has been hypothesized that TgCDPK3 controls rapid exit from the host by phosphorylating proteins needed for activating motility, the particular substrates of this kinase remained unknown. We have now applied an interaction trap system to identify the proteins that are modified by this kinase, which include a parasite motor protein Myosin A (TgMyoA). We show that TgCDPK3 specifically phosphorylates TgMyoA and this phosphorylation is important for parasite egress and motility.

## Introduction

The phylum Apicomplexa encompasses numerous obligate intracellular parasites that pose a significant health risk to animals and humans. Among these, *Toxoplasma gondii* is one of the most widespread, infecting all warm-blooded animals including approximately one third of the human population. Humans become infected congenitally or by ingestion of either environmental oocysts, which are shed in the feces of cats, or tissue cysts in the undercooked meat of infected animals. Most infections are asymptomatic during the acute stage but as to evade the immune response the parasite converts to a latent encysted form, thus establishing a chronic infection. In immunocompromised individuals and lymphoma patients, new infections or rupture of pre-existing cysts can lead to life-threatening toxoplasmic encephalitis [1–3]. Additionally, in congenital infections, toxoplasmosis can lead to blindness, severe neurological problems, or even death, given the immature nature of the fetal immune system [4].

A significant portion of the pathogenesis observed during toxoplasmosis is a direct consequence of the repeating cycles of invasion, division and egress that drive propagation of the parasite through the infected organism [5]. As the parasites escape their host cell during egress, the host membrane is ruptured resulting in cell death and an ensuing inflammatory response, both of which contribute to the pathogenesis of this infection. Active egress from the host cell involves parasite motility, cytoskeletal rearrangements within the parasite, and secretion from specialized organelles known as the micronemes [6–9]. A pore forming protein secreted from the micronemes, the perforin-like protein TgPLP1, facilitates egress by permeabilizing both the parasitophorous vacuolar membrane (PVM) and host plasma membrane [10]. Secretion of TgPLP1 and the initiation of motility during egress are regulated by calcium signaling, which is evident by the fact that treatment of intracellular parasites with calcium ionophores induces microneme secretion, motility and egress [6–9]. Calcium signaling in this parasite is quite distinct from what is typically observed in mammalian cells, involving plant-like factors such as the phytohormone abscisic acid (ABA) [11] and members of the family of Calcium Dependent Protein Kinases (CDPK) [12]. In particular, TgCDPK1 has been shown to be upstream of a signaling pathway regulating microneme secretion during egress and invasion [13].

Recently, three research teams, ours among them, identified a second calcium dependent protein kinase, TgCDPK3, as being critical for ionophore-induced egress (iiEgress) [14–16]. Through a series of selection and screens we isolated independent mutants that exhibit delayed iiEgress, resistance to extracellular exposure to calcium ionophores, which usually renders parasites non-invasive, and a reduction in *in vivo* virulence [17]. Whole genome sequencing of one of these mutant strains (MBE1.1) revealed a missense mutation that results in threonine for isoleucine (T239I) change within the catalytic domain of TgCDPK3 [18, 19]. As expected given the position of the mutated amino acid, this mutation significantly reduces the *in vitro* kinase activity of recombinant TgCDPK3 [14]. The critical role of TgCDPK3 as mediator of egress was validated when introduction of a wild type copy of TgCDPK3 was found to complement the phenotypes observed in MBE1.1.

Localization of TgCDPK3 to the periphery of the parasite [14] would suggest that it could phosphorylate membrane-associated proteins that influence egress, such as members of the motility machinery and those that regulate calcium signaling and fluxes. To experimentally determine the substrates of TgCDPK3 the relative phosphorylation site usage in wild type and *Tgcdpk3* mutant parasites was determined by quantitative mass-spectrometry using stable isotope labeling with amino acids in cell culture (SILAC) [20]. Comparisons of phosphorylation sites in wild type (WT) and mutant strains were made for intracellular parasites with and without ionophore. This analysis revealed 156 sites that are differentially phosphorylated between WT and mutant parasites. Importantly, most of the differential phosphorylation between the mutant and wild type strains is rescued in the complemented strain. A third of the phosphosites detected (51 of 156) showed a significant difference between WT and mutant parasites even in the absence of ionophore, indicating that TgCDPK3 regulates biological processes independent of iiEgress. This category includes proteins important for ion-homeostasis and metabolism, which is supported by the observation that basal calcium levels are increased in *Tgcdpk3* mutant parasites [20]. Among ionophore induced phosphosites that are more abundant in the WT than in the mutant strains are many that could play a role in egress or parasite motility such as Myosin A, F, and G, proteins of the inner membrane complex (IMC) [21] and a recently discovered protein that associates with cortical microtubules, TrxL-1 (TGGT1_115220) [22]. Interestingly a recent study showed that one of these candidates, Myosin A is phosphorylated in a calcium dependent manner at specific sites and that this phosphorylation event is important for parasite egress although the responsible kinase was not identified [23]. The list of proteins less phosphorylated in TgCDPK3 mutants also includes calcium-signaling proteins including a putative calmodulin (TGGT1_042450) and two calcium-dependent kinases (TgCDPK2a and TgCDPK3 itself). These results show that TgCDPK3 plays a pivotal role in regulating tachyzoite functions including, but not limited to, egress.

Given the complexity of the TgCDPK3-related phosphoproteome the mechanistic reason for the egress defect observed in parasites lacking TgCDPK3 function remains unexplained. In this study we define the TgCDPK3 interactome through the implementation of a proximity based interaction protein trap and identify Myosin A (TgMyoA) as a TgCDPK3 substrate. We show that TgCDPK3 specifically phosphorylates TgMyoA at Serines 21 and 743 *in vitro* and that these phosphorylation events are important for parasite egress *in vivo*.

## Results

### TgCDPK3-BirA* is targeted to the parasite plasma membrane and is functional

**Fig 1:**
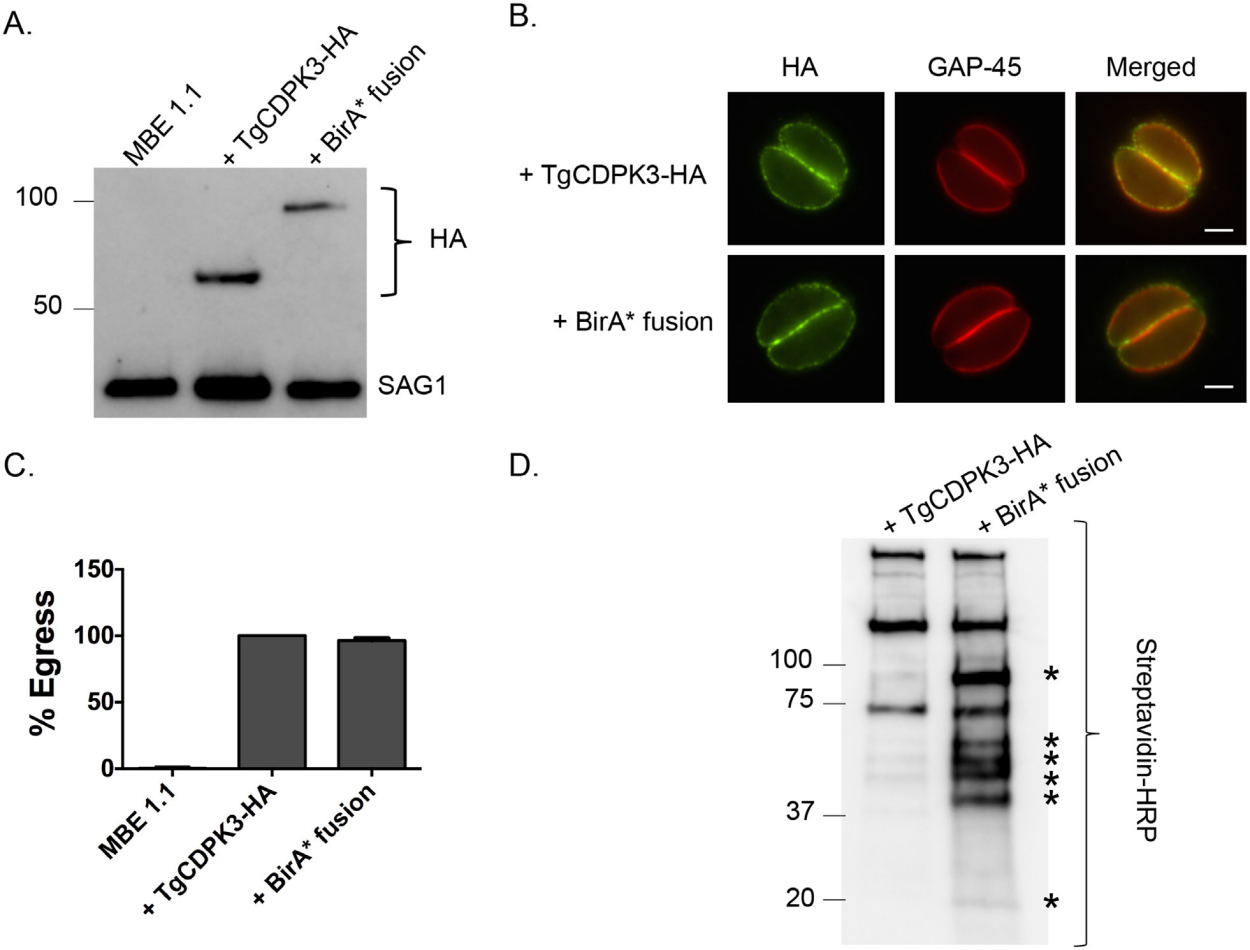
TgCDPK3-BirA* fusion protein is targeted to the plasma membrane and is functional. A. Western blot analysis of the *Tgcdpk3* mutant strain MBE1.1, or MBE1.1 complemented with either TgCDPK3-HA or TgCDPK3-BirA*-HA (BirA* fusion) using anti-HA antibody. The *Toxoplasma* protein SAG1 is used as loading control. B. Localization of TgCDPK3 and BirA* fusion protein in the *Tgcdpk3* mutant strain MBE1.1 was examined in intracellular parasites by IFA using anti-HA antibody. TgGAP45 is a marker for the inner membrane complex. Scale bar, 2 μM. C. Intracellular parasites of the MBE1.1 strain, or MBE1.1 complemented with either TgCDPK3-HA or the BirA* fusion were analyzed for egress efficiency by treating with the Ca^2+^ ionophore A23187 for 2 minutes. *n* = 3. Error bars, SEM. D. MBE1.1 parasites expressing either TgCDPK3-HA or TgCDPK3-BirA*-HA (BirA* fusion) were treated with biotin for 48 hours and biotinylated proteins were pulled down with streptavidin beads and analyzed by western using streptavidin-HRP antibody. Asterisks indicate proteins uniquely biotinylated in MBE1.1 + TgCDPK3-BirA*-HA parasites.

To identify putative substrates and interacting proteins of TgCDPK3 we utilized the BioID system, which relies on fusing a protein of interest to a mutant version of the bacterial BirA biotin ligase (BirA*) [24]. This mutant version of BirA lacks specificity and thus promiscuously biotinylates any protein within 10 nm of the fusion protein. Accordingly, we generated a construct in which BirA* is fused to the C-terminus of TgCDPK3 followed by a hemagglutinin (HA) epitope tag (TgCDPK3-BirA*-HA, aka BirA* fusion). The BirA* fusion construct was transfected into the *Tgcdpk3* mutant strain MBE1.1 [14]. As a control we transfected a construct carrying an HA tagged TgCDPK3 (TgCDPK3-HA) into MBE1.1 as well. Western blot using anti-HA antibodies showed that our recombinant strains correctly express either TgCDPK3-HA or TgCDPK3-BirA*-HA, both migrating at the expected size (Fig 1A). Immunofluorescence assays showed that the fusion protein is targeted to parasite periphery similarly to what is observed with TgCDPK3 (Fig 1B). Since we expressed the BirA* fusion protein in a strain lacking TgCDPK3 function we were able to test its functionality by its ability to complement the egress phenotype observed in *Tgcdpk3* mutant strains [14]. After 2 minutes of exposure to the calcium ionophore A23187, MBE1.1 parasites remained mostly intracellular (99.6%) while those expressing TgCDPK3-HA or BirA* fusion protein showed 100% and 96.4% egress respectively (Fig 1C). Thus, we have generated a strain expressing a BirA* fusion protein, which localizes correctly and is functional in the context of egress.

### TgCDPK3-BirA* biotinylates unique proteins within in the parasite

To identify putative TgCDPK3 interacting proteins we grew TgCDPK3-HA and BirA* fusion-expressing parasites in the presence of biotin. Lysates of both cultures were treated with RIPA buffer and the supernatant was subjected to affinity purification with streptavidin conjugated magnetic beads to trap the biotinylated proteins. Western blot of the precipitated material showed that, in addition to proteins that were common between the TgCDPK3-HA and BirA* fusion protein expressing parasites, there were several proteins that appeared to be biotinylated solely in the BirA* fusion protein expressing parasites (Fig 1D). Having confirmed the presence of various proteins exclusively biotinylated in the parasites expressing BirA* fusion protein, we scaled up the affinity purification of biotinylated proteins from parasites grown with biotin and subjected the resulting material to mass spectroscopy analysis. This analysis identified six proteins that were common between the two strains (S2 Table) and fourteen proteins that were detected only in the MBE1.1 + TgCDPK3-BirA* parasite sample including TgCDPK3, which was expected as the BirA* fusion would biotinylate itself (Table 1). Remarkably, seven of the proteins identified through our approach were previously shown through a proteomic study to be differentially phosphorylated between wild type and *Tgcdpk3* mutant parasites (Table 1, in bold). Having identified these proteins through two independent approaches strongly suggests that they might be direct substrates of TgCDPK3.

**Table 1:** Proteins uniquely biotinylated in BirA* fusion protein expressing parasites. In bold are those that were previously determined to be differentially phosphorylated in strains lacking TgCDPK3 function (20).

ID	Description	No. of Peptides
**TGGT1_235470**	**Myosin A**	**13**
TGGT1_280410	3'5'-cyclic nucleotide PDE domain-containing protein	4
TGGT1_249900	Adenine nucleotide translocator, putative	7
**TGGT1_206590**	**CDPK2A**	**3**
TGGT1_223940	GAP45	4
**TGGT1_289690**	**GAPDH1**	**2**
TGGT1_212090	Hypothetical protein	4
TGGT1_310420	Hypothetical protein	1
**TGGT1_289760**	**Hypothetical protein**	**3**
**TGGT1_313290**	**MORN repeat-containing protein**	**2**
**TGGT1_314780**	**Myosin G**	**5**
TGGT1_232340	Protein phosphatase 2C domain-containing protein	8
**TGGT1_231630**	**IMC4**	**1**

### TgCDPK3 phosphorylates S21 and S743 of TgMyoA

**Fig 2:**
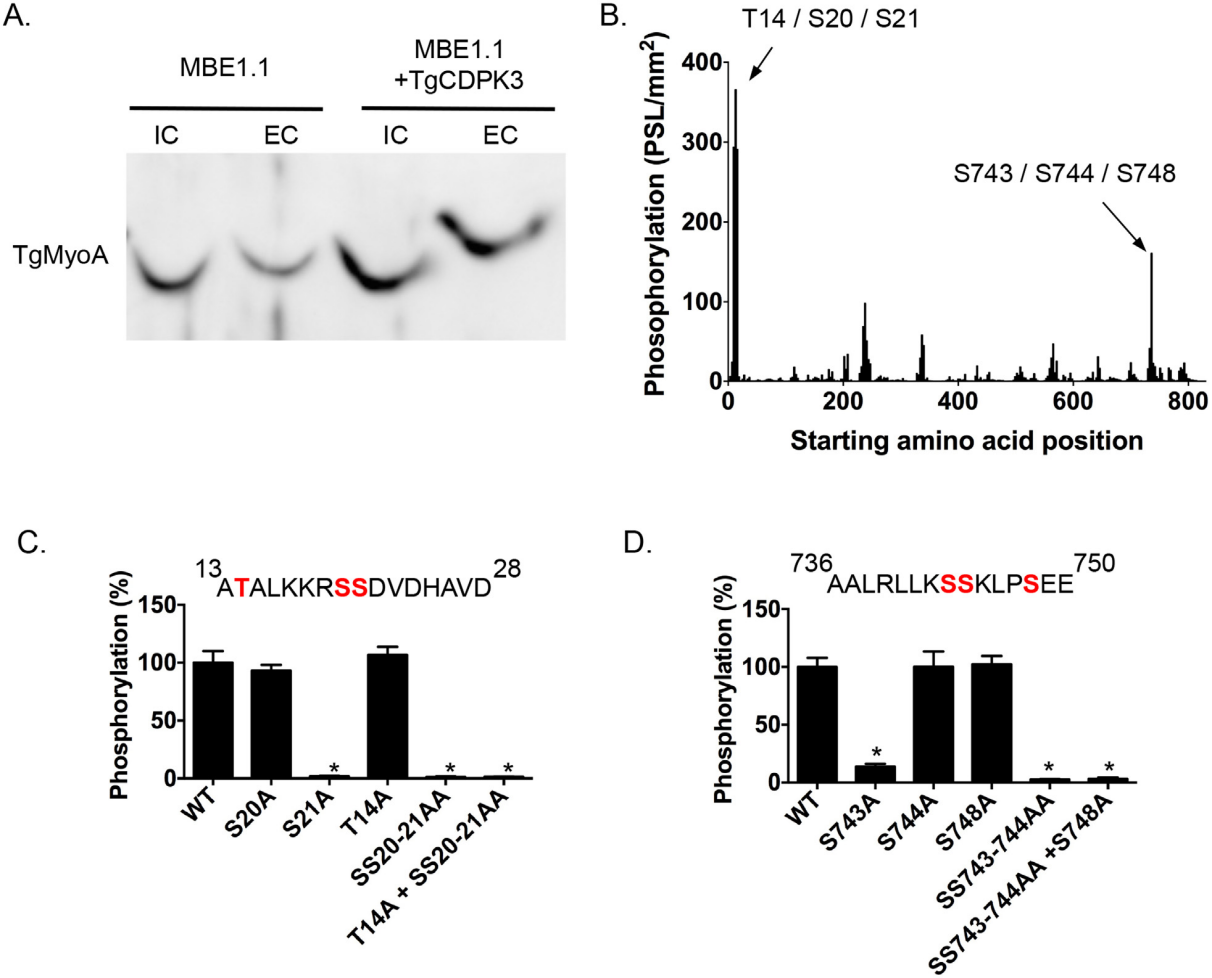
TgCDPK3-dependent phosphorylation of TgMyoA *in vivo* and *in vitro*. A. Phosphorylation status of TgMyo-A in MBE1.1 and MBE1.1 + CDPK3-WT parasites was analyzed using Phos-tag gel electrophoresis and Western blot using antibody against TgMyoA. Parasites were manually extracted from host cells and incubated in either intracellular (IC) or extracellular (EC) buffer for 2 minutes. B. Mapping of TgCDPK3 phosphorylation sites on TgMyoA by tiled peptide array analysis using purified recombinant TgCDPK3. Phosphorylation intensity of 15 amino acid length peptides that span full-length TgMyoA and are each shifted by 3 amino acid was detected using MultiGauge version 3.0. The serines and threonines in the two peptides that showed phosphorylation signal more than 100 PSL/mm^2^ are indicated above the corresponding peaks. Fine mapping of TgCDPK3 phosphorylation sites on TgMyoA is shown in C and D. Phosphorylation by recombinant TgCDPK3 was tested on peptides that contained single, double and triple mutations of T14, S20 and S21 residues in the peptide ^13^ATALKKRSSDVDHAVD^28^ (C) and S743, S744 and S748 residues in the peptide ^736^AALRLLKSSKLPSEE^750^ (D) *n* = 3, Error bars, SEM. (*P < .05, students *t* test).

Among the proteins that interact with TgCDPK3-BirA* the top hit was Myosin A (TgMyoA), which was also identified as less phosphorylated at serine 20 or 21 in the phosphroproteome of parental and *Tgcdpk3* mutant parasites [20]. To further confirm that TgMyoA is less phosphorylated in *Tgcdpk3* mutant parasites we performed Phos-tag gel electrophoresis, which involves use of Phos-tag biomolecule that specifically binds phosphorylated proteins and retards their migration in the gel [25]. Towards this goal, we harvested intracellular MBE1.1 (*Tgcdpk3* mutant) or MBE1.1+TgCDPK3-HA parasites in presence of intracellular buffer and re-suspended in either intracellular or extracellular buffer followed by incubation at 37°C for 2 minutes and examined the phosphorylation status of TgMyoA (Fig 2A). The results showed that TgMyoA’s migration is significantly slower in extracellular conditions, indicating that it is phosphorylated when parasites transition from intra- to extra-cellular conditions. Importantly, this shift in migration of TgMyoA is reduced in the *Tgcdpk3* mutant strain, MBE1.1, confirming that TgMyoA is less phosphorylated in the absence of TgCDPK3 function (Fig 2A).

As a next step we set out to determine whether TgCDPK3 can phosphorylate TgMyoA and TgGAP45, another protein that forms part of *T*. *gondii*’s motility complex [7], and was also exclusively identified in the sample from biotin exposed TgCDPK3-BirA* parasites. Towards this goal we performed an *in vitro* phosphorylation assay using purified recombinant TgCDPK3 and a non-biased overlapping peptide array covering the entire TgMyoA and TgGAP45 sequences. Each peptide was 15 amino acids in length and tiled peptides were shifted by 3 amino acids. The peptides (273 for TgMyoA and 78 for TgGAP45) were spotted on a modified cellulose membrane using routine Fmoc (N-(9-fluorenyl)methoxycarbonyl) chemistry, deprotected and exposed to activated recombinant TgCDPK3 in presence of [γ-^32^P]ATP and calcium. Peptide spots phosphorylation was quantified using phosphoimaging. For TgMyoA, two peptides (^13^ATALKKRSSDVDHAVD^28^ and ^736^AALRLLKSSKLPSEE^750^) showed phosphorylation signal >100 PSL/mm^2^ (Fig 2B). By contrast, none of the peptides spanning GAP45 showed significant phosphorylation signal (S1 Fig). TgCDPK3 is a serine threonine kinase and in each of the two peptides of TgMyoA that were phosphorylated there are 3 potential phosphorylation sites (Fig 2B). To determine the specific residues that are phosphorylated we generated mutated versions of both peptides that contained single, double or triple mutations where serine (S) or threonine (T) were mutated to the non-phosphorylable residue alanine. *In vitro* phosphorylation of these mutant peptides with purified recombinant TgCDPK3 showed that in the peptide ^13^ATALKKRSSDVDHAVD^28^, mutation of either T14 or S20 does not affect phosphorylation signal while mutation of S21 results in complete loss of phosphorylation (Fig 2C). In the second peptide ^736^AALRLLKSSKLPSEE^750^ mutation of S744 or S748 does not significantly affect phosphorylation while mutation of S743 leads to 96.4% loss of phosphorylation signal (Fig 2D). These results indicate that TgCDPK3 can specifically phosphorylate S21 and S743 residues of TgMyoA.

Previous studies have shown that in *T*. *gondii*, TgMyoA is phosphorylated at multiple sites including S21 [20, 26]. However, TgMyoA S743 has not been previously reported as phosphorylated in *Toxoplasma* parasites. To address whether S743 is phosphorylated *in vivo* we immuno-precipitated the motor complex with an antibody against TgGAP45 and analyzed the phosphorylation status of TgMyoA by mass spectrometry. The analysis indicated that S743 is indeed phosphorylated in intracellular parasites as evidenced by phosphorylation status of the first serine of the peptide ^743^SSKLPSEEYQLGKTMVFLK^760^ (S2 Fig).

### Phosphorylation of S21 and S743 is important for parasite egress

**Fig 3:**
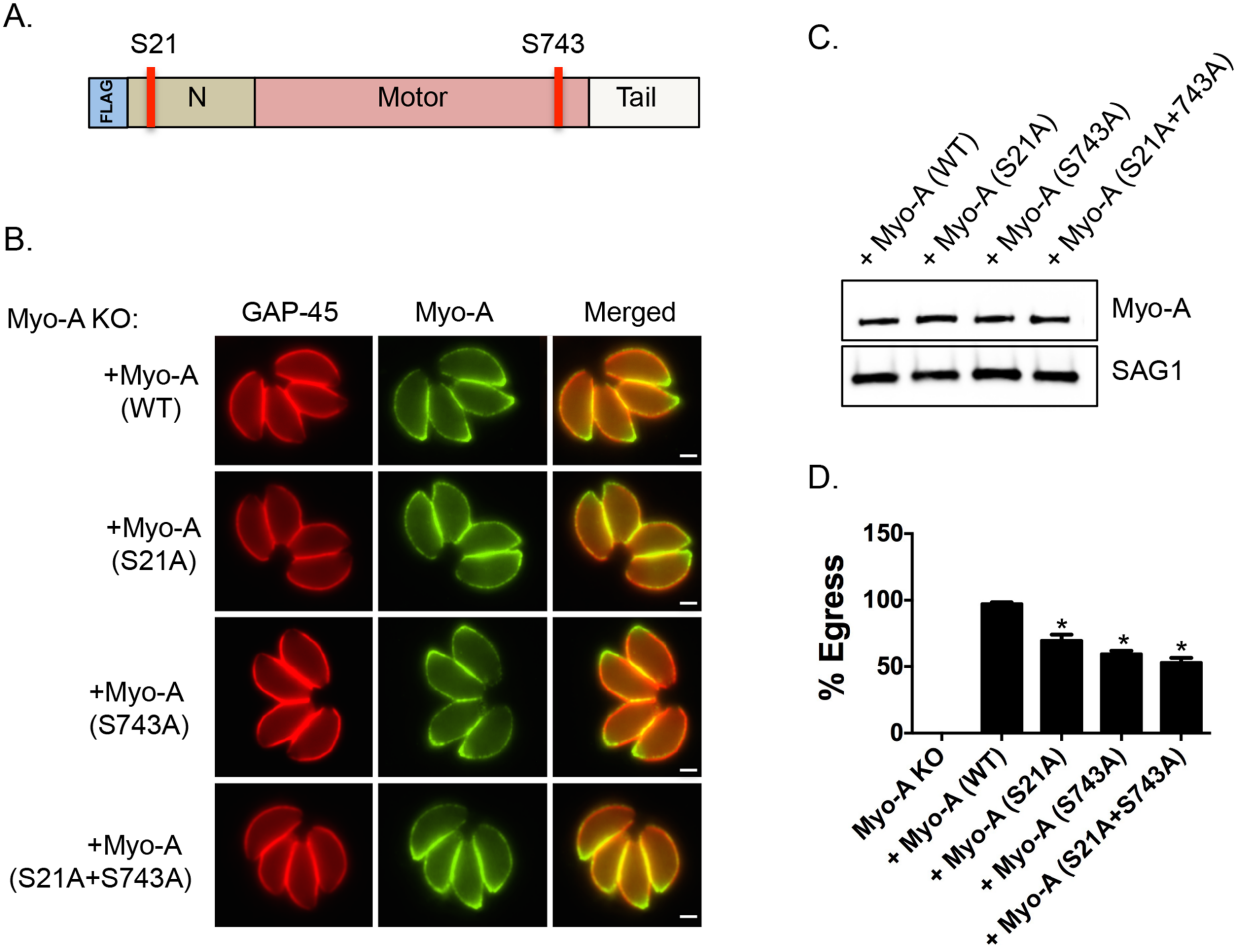
Phosphorylatable serines 21 and 743 of TgMyoA are important for iiEgress of *Toxoplasma* tachyzoites. A. Schematic representation of TgMyoA with relative location of S21 and S743. B. TgMyoA null mutants were complemented with Flag-tagged wild type TgMyoA or Flag-tagged mutant versions of TgMyoA. Localization of the Flag tagged transgenic TgMyoA was examined in intracellular parasites by IFA using anti-Flag antibody. TgGAP45 is used as a marker for the IMC. Scale bar, 2 μM. C. Western blot analysis of TgMyoA null mutants complemented with either wild type or the mutant TgMyoA was performed using anti-Flag antibody. SAG1 is used as loading control. D. Intracellular parasites of the TgMyoA null strain as well as those complemented with either wild type TgMyoA or S21A, S743A or S(21+743)A mutant TgMyoA were analyzed for egress efficiency by treating with the Ca^2+^ ionophore A23187 for 2 minutes. *n* = 3. Error bars, SEM. (*P < .05, students *t* test).

Interestingly, it has been previously reported that genetic disruption of TgMyoA results in a delay of ionophore-induced egress reminiscent of what is observed in *Tgcdpk3* mutant parasites [9]. To determine the importance of phosphorylation of S21 and S743 of TgMyoA during induced egress, a process that is regulated by TgCDPK3, we complemented a TgMyoA null mutant strain with either wild type TgMyoA or TgMyoA in which either S21 or S743, or both were mutated to alanine (Fig 3A). Immunofluorescence assays of parasites expressing the wild type or mutant MyoA indicate that the transgenic proteins correctly localize to the inner membrane complex (Fig 3B). Importantly, western blot analysis showed that wild type and mutant TgMyoA are expressed at similar levels in these transgenic parasites (Fig 3C). We exposed these transgenic parasite lines as well as the TgMyoA knockout strain (MyoA KO) to A23187 for 2 minutes to determine the efficiency of ionophore-induced egress. As expected, the TgMyoA KO exhibited a strong egress defect (1% egress), which was complemented by expression of wild type TgMyoA (97.2% egress, Fig 3D). By contrast, the TgMyoA mutants TgMyoA(S21A), TgMyoA(S743A), and TgMyoA(S21A+S743A) only partially rescued the egress phenotype with 69.8%, 59.5%, and 53% egress, respectively (Fig 3D). These results suggest that the presence of a phosphorylatable serine at positions 21 and/or 743 of TgMyoA contributes to *Toxoplasma* egress from host cells.

### Phosphomimetic mutants of TgMyoA compensate lack of TgCDPK3 function

**Fig 4:**
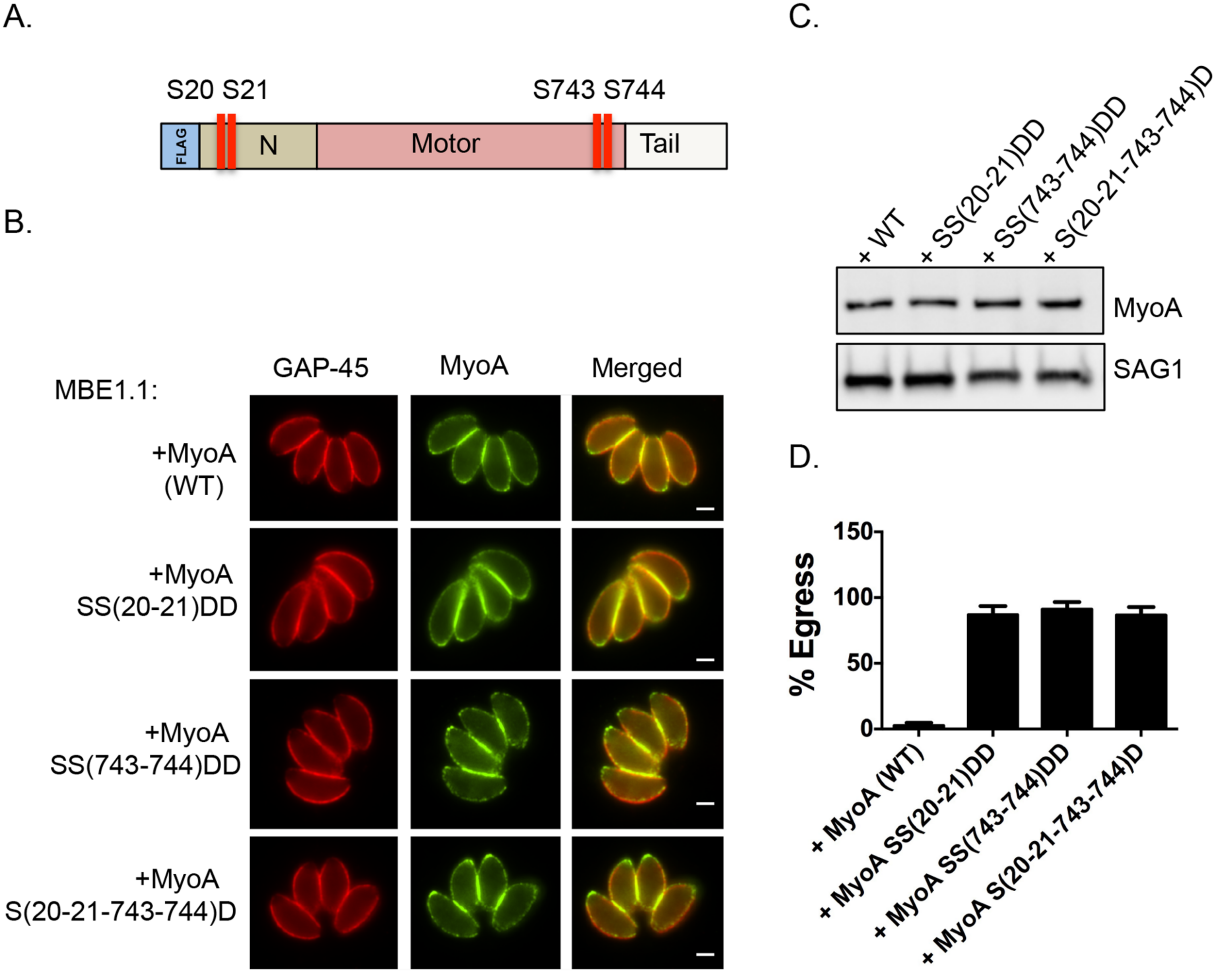
Constitutive phosphorylation of TgMyoA negates the requirement for TgCDPK3 during egress. A. Schematic representation of TgMyoA with relative location of S20, S21 S743 and S744. IFA (B) and Western blot analysis (C) of Flag tagged TgMyoA in MBE1.1 (*Tgcdppk3* mutant) parasites complemented with either wild type TgMyoA or SS20-21DD, SS743-744DD or S(20,21,743,744)D mutant TgMyoA was performed using anti-Flag antibody. TgGAP45 is used as a marker for IMC in the IFAs and SAG1 is used as loading control in Western blot. Scale bar, 2 μM. D. Intracellular parasites were analyzed for egress efficiency by treating with the Ca^2+^ ionophore A23187 for 6 minutes. *n* = 3. Error bars, SEM.

We next tested whether mutating S21 and/or S743 of TgMyoA to the phosphomimetic residue aspartic acid could rescue the egress defect of *Tgcdpk3* mutant parasites. Because phosphomimetic residues (aspartic acid or glutamic acid) do not fully approximate the electronegativity produced by phosphorylation, we employed the strategy of mutating two neighboring pairs of amino acids to overcome the charge differential [27, 28]. Accordingly, we transfected the *Tgcdpk3* mutant strain MBE1.1 with either a FLAG tagged wild type copy of TgMyoA or FLAG tagged TgMyoA in which serine residues 20 and 21, 743 and 744, or 20, 21, 743 and 744 were mutated to aspartic acid (Fig 4A). Immunofluorescence assays and Western blots assays indicated that all versions of TgMyoA were correctly targeted and expressed at equal levels (Fig 4B and 4C). At 2 minutes of exposure to A23187, which is sufficient to induce egress of 100% of wild type parasites (Fig 1C), MBE1.1 mutant parasites expressing wild type or phosphomimetic MyoA showed only 0.4% and 3.6% egress respectively. Nonetheless, by 6 minutes of ionophore treatment we saw a significant difference between the MBE1.1 mutant parasites expressing an exogenous copy of wild type MyoA and those expressing the phosphomimetic versions of the protein (Fig 4D). Induction of egress with A23187 for 6 minutes showed that nearly all (97.3%) MBE1.1 parasites expressing the exogenous wild type copy of MyoA remained inside of the cells after six minutes of treatment. This indicates that overexpression of TgMyoA does not rescue the egress defect associated with lack of TgCDPK3 activity. By contrast, expression of either TgMyoA SS(20–21)DD, TgMyoA SS(743–744)DD and TgMyoA S(20-21-743-744)D in MBE1.1 significantly complemented the ionophore induce egress phenotype (87%, 91.3%, and 86.7% egress at 6 minutes post induction respectively, Fig 4D). Thus, mimicking constitutively phosphorylated TgMyoA partially overrides the need for TgCDPK3 function during calcium-stimulated egress.

### Phosphorylation of S21 and S743 of TgMyoA is important for parasite motility

**Fig 5:**
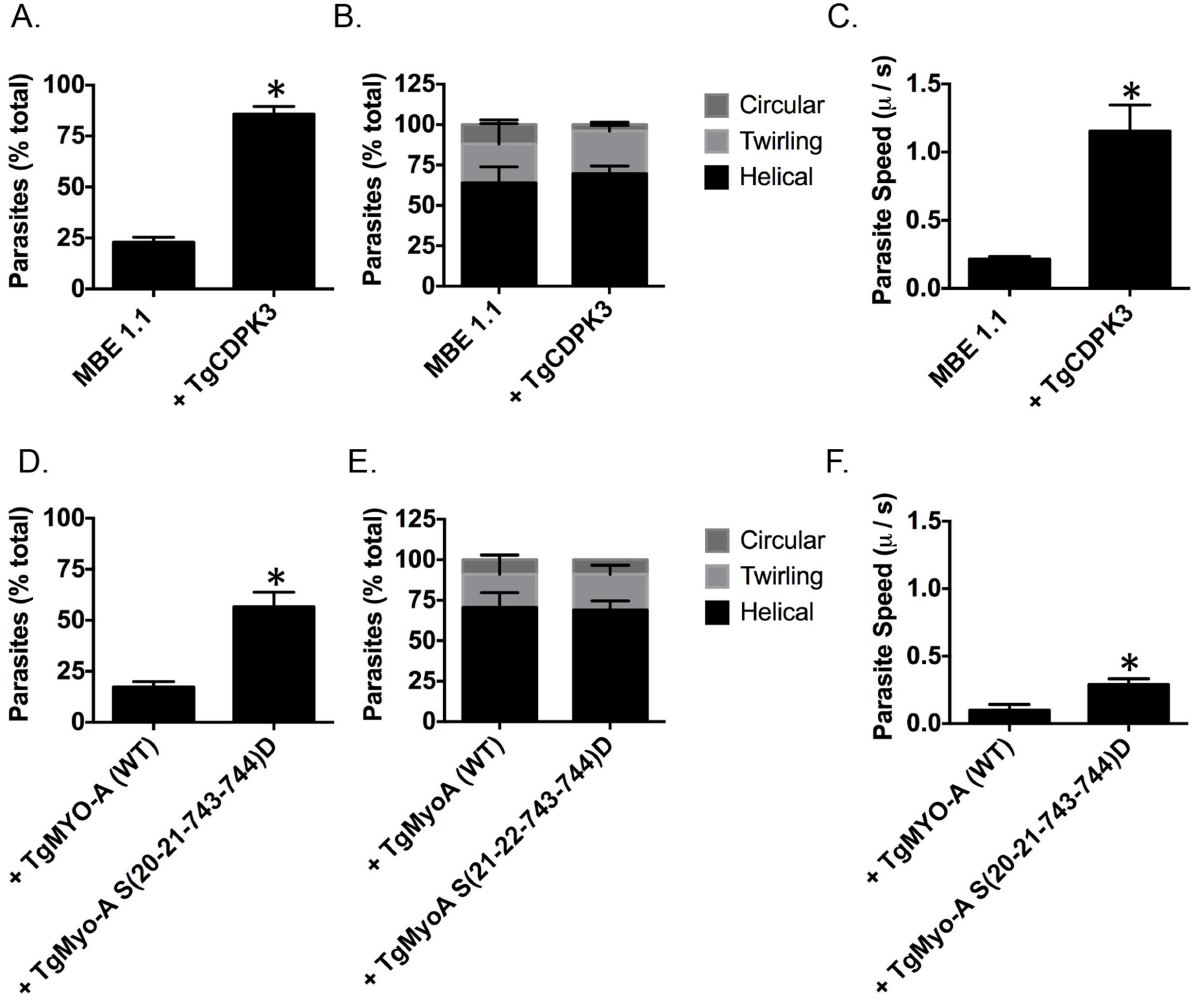
TgCDPK3 is required for initiation of motility. Quantification of parasite motility by 2D live video microscopy when parasites are switched from intracellular buffer to extracellular buffer condition (A and D). Parasite motility is shown as the mean percentage of total number of parasites that exhibited motility or no motility. Quantification of different forms of motility exhibited by parasites when parasites are switched from intracellular buffer to extracellular buffer condition (B and E). Parasite motility is shown as the mean percentage of total number of motile parasites that exhibited motility (helical, twirling and circular gliding). Speed of parasite gliding when parasites are switched from intracellular to extracellular conditions (C and F). n = 3, Error bars = SEM. (*P < .05, students *t* test).

TgMyoA is an important component of glideosome and plays a critical role in parasite motility [29, 30]. Thus, it is plausible one of the roles of TgCDPK3 during induced egress is to initiate motility via the phosphorylation of TgMyoA. Interestingly, previous studies have shown that chemical inhibition of TgCDPK3 affects initiation of motility in extracellular parasites [15]. Additionally, we have previously shown that *Tgcdpk3* mutant strains have reduced efficiency of invasion [14, 17], a process that depends on motility. To further examine the role of TgCDPK3 in parasite motility, we tested the efficiency of the *Tgcdpk3* mutant strain (MBE1.1) and the complemented strain (MBE1.1+TgCDPK3) in transitioning from a non-motile to a motile state. This was accomplished by recording and analyzing live video microscopy of parasites for two minutes after changing the media from one that mimics intracellular conditions (IC buffer) to one that mimics extracellular conditions (EC buffer) [31]. While 85.8% of the complemented parasites had become motile by two minutes after switching the media, only 22.8% of the TgCDPK3 mutant parasites were motile during the same time period (Fig 5A). *Toxoplasma* parasites normally exhibit three types of motility patterns referred to as helical, twirling and circling [32]. Therefore, we scored the type of motility exhibited by those parasites of either strain that were moving to determine whether TgCDPK3 played a role in a specific type of movement. The results showed that the proportion of parasites exhibiting each type of movement was similar between the two strains (Fig 5B). As we noted that the mutant parasites appear to move at a slower pace than wild type ones, we also examined speed of parasite movement when switched from intracellular to extracellular conditions. TgCDPK3 mutants moved with an average speed of 0.21 μ/s while complemented parasites exhibited a much higher speed of 1.15 μ/s (Fig 5C). Therefore, the large difference between the mutant and complemented strains in the percentage of parasites that quickly initiated motility and also faster speeds confirm that TgCDPK3 plays a role in parasite motility.

We next wanted to determine if phosphomimetic mutants of TgMyoA could rescue the motility defect in TgCDPK3 mutant parasites. For this we analyzed MBE1.1 parasites expressing an extra copy of either TgMyoA (WT) or TgMyoA S(20-21-743-744)D by video microcopy. The analysis showed that only 17.3% of MBE1.1 + TgMyoA (WT) parasites became motile once transitioned from IC to EC buffer (Fig 5D). By contrast, MBE1.1 parasites complemented with TgMyoA S(20-21-743-744)D showed increased levels of motility initiation with 56.6% of them becoming motile during the first two minutes after switching the buffer (Fig 5D). Quantification of three kinds of gliding motility between the two strains showed that the proportion of each movement was similar between the two strains (Fig 5E). However, when examined for speed, MBE1.1 + TgMyoA (WT) showed a pace of 0.1 μ/s while MBE1.1 + TgMyoA S(20-21-743-744)D moved at a slightly higher speed of about 0.29 μ/s (Fig 5F). These results indicate that phosphomimetics of TgMyoA can rescue the motility defect of *Tgcdpk3* mutants at least in number of motile parasites suggesting they can compensate lack of TgCDPK3 function in initiating parasite motility. However, as it is the case for iiEgress, this rescue is not complete, as the speed of the MBE1.1 + TgMyoA S(20-21-743-744)D parasites is still approximately threefold less than the wild type parasites.

## Discussion

Although it is well established that TgCDPK3 is important for parasite egress, the particular mechanism by which this calcium-stimulated kinase regulates this key event of the lytic cycle is not known. A recent study revealed 156 phosphorylation sites out of more than 12,000 quantified that were differentially phosphorylated between wild type and *Tgcdpk3* mutant parasites, with many of them related to motility, ion-homeostasis and metabolism [20]. While some of these differentially phosphorylated sites might be direct substrates of TgCDPK3, one might expect that a number of these sites related to downstream effects and compensatory mechanisms related to a loss in TgCDPK3 signaling. In addition, TgCDPK3 is involved in several processes such as calcium homeostasis and parasite division, which might involve distinct substrates from those involved in egress. Therefore, additional efforts were needed to identify the specific protein(s) whose phosphorylation by TgCDPK3 is key for induced egress and initiation of motility. With this in mind we successfully adapted the BioID system to identify putative substrates and interactors of TgCDPK3. This approach, which is based on fusing the protein of interest to a promiscuous allele of the biotin ligase BirA, has the advantage that it can identify not only direct interactors but also proteins that are nearby or interact loosely or transiently, such as enzyme substrates. Identification by proximity labeling does not prove an enzyme/substrate relationship, and it is plausible that some of these interactions are structural in nature. However, in combination with our previous phosphoproteome analysis [20], which identified seven of the thirteen proteins identified through BioID as less phosphorylated in *Tgcdpk3* mutant parasites, it provides important indirect evidence for a kinase/ substrate relationship. Having been linked to TgCDPK3 in two independent and distinct approaches makes these seven proteins strong candidates for being TgCDPK3 substrates during the events regulated by this kinase.

These seven putative substrates include TgCDPK2a, GAPDH1, a MORN repeat containing protein, IMC4, TgMyoA and TgMyoG, and a hypothetical protein of unknown function. Interestingly, many of these proteins are known or would be predicted to be within the periphery of the parasite, which strengthens the argument that they might be TgCDPK3 substrates. For example, IMC4 is part of the inner membrane complex [33], which is a continuous layer of flat vesicles sutured together and to which the motor protein TgMyoA is anchored [30]. The glyceraldehyde 3-phosphate dehydrogenase 1 (TgGAPDH1), which is normally cytoplasmic, redistributes to the periphery of the parasite during egress [31]. Additionally, both the MORN repeat-containing protein and the hypothetical proteins (TgGT1_310420) are predicted to be myristoylated, which suggests membrane localization. The function of the hypothetical protein is not known, but MORN proteins are involved in cell division in eukaryotes including *T*. *gondii* [34, 35]. Interestingly, among the putative substrates identified in both the proteome and the BioID approaches, is a second calcium dependent protein kinase (TgCDPK2a), which suggests that these kinases might work together as co-regulators of a protein network or as part of a signaling cascade. Nonetheless, at present no information as to either the localization or the function of TgCDPK2a is available.

Of special interest among the proteins identified through BioID are TgMyoA and GAP45, both of which form part of the motor complex driving the parasite’s gliding motility. The so-called glideosome resides in the space between the parasite plasma membrane and the IMC and it is a complex of several proteins including TgMyoA, two associated light chains, myosin light chain TgMLC1 and essential light chain TgELC1 and the glideosome associated proteins TgGAP40, TgGAP50, TgGAP45 or TgGAP70 [7, 30, 36, 37]. Given the proximity of TgCDPK3 to the glideosome and the facts that induced egress is dependent on motility and that motility is a calcium-dependent process, a functional connection between TgCDPK3 and the motility machinery is a plausible one. Recent studies using a small molecule invasion enhancer that causes an increase in intracellular Ca^2+^ showed that TgMyoA is phosphorylated in a calcium dependent manner at specific residues, serine 20, 21 and 29 and that phosphorylation of serine 21 is important for ionophore induced egress and motility [26]. However the kinase that mediates this phosphorylation process had not been known. Interestingly, TgCDPK3 had been considered as a likely candidate given the remarkably similar egress phenotype seen in both the *TgmyoA* and *Tgcdpk3* mutant strains. Consistent with this idea peptides containing phosphorylated serine 20 or 21 were found to be less abundant in *Tgcdpk3* mutant strains as compared to the parental or complemented ones in proteomic studies [20]. This finding suggested that the phosphorylation status of TgMyoA, at least for Ser20/21 may be coupled to TgCDPK3 signaling. Our BioID and mutagenesis data suggests that TgMyoA is directly regulated by TgCDPK3 and is thus a *bona fide* substrate for TgCDPK3.

Our data strongly argues for a direct relation between TgMyoA phosphorylation and TgCDPK3. Based on our BioID results, TgMyoA either interacts with or is in close proximity to TgCDPK3. Consistent with this idea; we show that recombinant TgCDPK3 can indeed phosphorylate TgMyoA *in vitro* with preference for serines 21 and 743. While CDPKs can act non-specifically *in vitro*, it is important to note that we did not detect significant phosphorylation of any of the other known phosphorylated amino acids of TgMyoA and of none of those from TgGAP45, indicating that we are observing some level of specificity in our peptide array assay. Interestingly, TgGAP45 was not observed as less phosphorylated in our mutant strains [20]. Therefore, any interaction between TgCDPK3 and TgGAP45 is likely to be structural and not enzymatic and the phosphorylation state of TgGAP45, which is important for its function [38], is likely regulated by a different kinase.

Creating a version of TgMyoA that ‘looks’ phosphorylated overrides the need for TgCDPK3, strongly indicating that this is the kinase responsible for modifying TgMyoA. Nonetheless, the complementation of iiEgress by the phosphomimetic versions of TgMyoA is partial. While the levels of egress and motility exhibited by the phosphomimetic expressing strains are significantly higher than that of the CDPK3 mutant strain, they don’t reach wild type levels. There are several plausible reasons for this incomplete complementation of the iiEgress phenotype, including the fact that phosphomimetic mutations are not a perfect simulation of phosphorylated serine [27, 28]. Also, phosphomimetic mutations that result in constitutively active serine or threonine do not allow for dynamic changes of alternating phosphorylation and dephosphorylation events that might be occurring *in vivo* and are important for function. Another possibility we must consider is that other amino acids within TgMyoA are also regulated by TgCDPK3, but were not revealed in our *in vitro* assays. Finally, and most likely, TgMyoA might not be the only substrate through which TgCDPK3 is exerting its regulation of iiEgress and/or other kinases might work redundantly along with TgCDPK3. We have previously shown that disruption of TgCDPK3 results in dysregulation of calcium homeostasis, a phenotype not observed previously with any TgMyoA mutants, which could affect sensitivity to the ionophore. We haven’t specifically tested whether TgMyoA has an effect on resting calcium levels in this study but its involvement is unlikely given its predicted role as part of the molecular motor that drives movement of the parasite. Several of the putative substrates we identified including TgCDPK2A, Myosin-G and GAPDH1 are good candidates for influencing egress and future work will focus on understanding their potential contribution to TgCDPK3 regulated events.

An interesting question that remains unanswered is the particular timing of the phosphorylation of TgMyoA by TgCDPK3. Does TgCDPK3 phosphorylate TgMyoA during intracellular growth or does it occur upon induction of egress? Based on the results obtained using Phos-tag (Fig 2A) it appears that there is a significant level of TgCDPK3-dependent phosphorylation of TgMyoA upon the transition from intracellular to extracellular conditions, which mimics what the parasite encounters during egress. Nonetheless, based on phosphoproteomic comparison between wild type and *Tgcdpk3* mutant parasites, phosphorylated Ser21 is more abundant in the wild type strain even in intracellular parasites not exposed to ionophore, which would suggest this phosphorylation event occurs in the absence of egress induction. Interestingly, it has been reported that phosphorylation of several amino acids in TgMyoA is dependent on Ser21 being phosphorylated first [26]. Thus, a plausible model that would explain these various results is that TgCDPK3 phosphorylates TgMyoA at Ser21 in response to calcium fluxes that occur during intracellular growth, and that upon induction of egress either TgCDPK3 or another kinase further phosphorylates TgMyoA in a phospho-Ser21 dependent manner. Thus, in the absence of TgCDPK3 phosphorylation of TgMyoA is significantly altered during egress due to lack or reduction of Ser21 phosphorylation.

Another important standing question is how phosphorylation of TgMyoA at those two particular sites influences its function at the mechanistic level. The importance of phosphorylation is well established for class II myosins, which are found in skeletal muscle. Nonetheless, TgMyoA is quite divergent structurally from other myosins [39, 40] and therefore its regulation is likely to be unique. TgMyoA is a single headed motor protein [41] that belongs to the class XIVa myosin family which is unique to Apicomplexans and ciliates and the conserved motor domain shares only about 23–34% homology with mammalian myosins [23, 39]. Class XIVa myosins also lack the conserved glycine at the lever arm pivot point and have a shorter C-terminal tail, which has been shown to be important for motor function in class II myosins [41]. In TgMyoA, Ser 21 is located in the N-terminal region whose role remains undefined, while Ser 743 lies within the motor domain. It is feasible that phosphorylation of these residues either results in structural modification of TgMyoA that in turn allows new protein-protein interactions or activates its enzymatic activity both of which could be important for mechanochemical function of TgMyoA. The recent successful expression and purification of recombinant TgMyoA [42] will be particularly useful to investigate how phosphorylation influences the function and biochemistry of this unique and key motor protein. Those new *in vitro* methods along with our novel discovery that TgCDPK3 phosphorylates TgMyoA within the parasite to initiate egress, will provide a more complete understanding of how motility is tightly regulated during the lytic cycle of this important human parasite.

## Materials and Methods

### Parasite cultures

*Toxoplasma gondii* tachyzoites were maintained by passage through human foreskin fibroblasts (HFF, obtained from the American Tissue Culture Collection ATCC) in a humidified incubator at 37°C with 5% CO_2_. Normal growth medium consisted of DMEM supplemented with 10% fetal bovine serum, 2 mM L-glutamine and 50μg/ml of penicillin-streptomycin. Purification of parasites was performed as previously described [43].

### Plasmid constructs

Primers used in generating plasmid constructs described in this section are listed in supplemental table S1 (S1 Table). To generate the BirA* fusion (TgCDPK3-BirA-HA), CDPK3-BirA*-HA was commercially synthesized (GenScript, USA), amplified by PCR using specific primers (S1 Table) and directionally cloned downstream of the *Tgcdpk3* promoter in the vector, pTgcdpk3CDPK3-HA [43] using *Nco*I and *Pac*I sites. The non-phosphorylable and phosphomimetic mutants of TgMyoA were made using Lightning site directed mutagenesis kit (Agilent Technologies) with primers listed in S1 Table and pmyoA-FLAGTgMyoA-WT/graBle [23] as the parent plasmid. All resulting constructs were verified by restriction digestion and sequencing.

### Stable transfection

Plasmid constructs were linearized with the restriction enzyme KpnI, purified and electroporated into *T*. *gondii* tachyzoites according to established protocols [44, 45]. Parasites transfected with BirA* fusion construct were cultured in presence of 50 μg/ml mycophenolic acid (MPA) and 50 μg/ml xanthine and cloned by limiting dilution to obtain stably transformed clones. When using vectors carrying the Ble gene as a selectable marker, transfected parasites were added onto an HFF monolayer and allowed to grow without any drug selection until the monolayer was lysed. Freshly egressed parasites were then washed with Hanks’s balanced salt solution containing 10 mM HEPES and 0.1 mM EGTA (HHE) and extracellular parasites were treated with 50 μg/ml phleomycin in DMEM for 4 hours at 37°C with 5% CO_2_. The parasites were then added onto a HFF monolayer and cultured in the presence of 5 μg/ml phleomycin to select drug resistant parasites, which were cloned by limiting dilution.

### Affinity purification of biotinylated proteins

Affinity purification of biotinylated proteins was performed according to previously described protocols with minor modifications [24, 46]. Briefly, parasites were cultured in growth medium containing biotin (150 μg/ml) for 48 hours. Freshly egressed parasites (2.5 x 10^9^) were then washed with phosphate buffered saline (PBS) and lysed with 1 ml RIPA buffer (20 mM Tris-HCl (pH 7.5), 150 mM NaCl, 1 mM Na_2_EDTA, 1 mM EGTA, 1% NP-40, 1% sodium deoxycholate, 2.5 mM sodium pyrophosphate, 1 mM β-glycerophosphate, 1 mM Na_3_VO_4_) supplemented with complete protease inhibitor (Roche) and centrifuged at 16000 g for 15 minutes at 4°C. The supernatant was then transferred to a fresh tube and incubated with magnetic streptavidin beads (Dynabeads Myone streptavidin C1 from Invitrogen) at 4°C for 12 hours with gentle shaking. Beads were then collected with magnets and washed twice with wash buffer 1 (2% SDS), once with wash buffer 2 (0.1% deoxycholate, 1% Triton X-100, 500 mM NaCl, 1 mM EDTA and 50 mM HEPES, pH 7.5), once with wash buffer 3 (250 mM LiCl, 0.5% NP-40, 0.5% deoxycholate, 1 mM EDTA and 10 mM Tris pH 8.1), twice with wash buffer 4 (50 mM Tris, pH7.4 and 50 mM NaCl) and twice with PBS, in that order. The beads were finally re-suspended in 1 ml PBS and 10% of each sample was then boiled at 98°C for 5 minutes to separate bound proteins from magnetic beads and eluted proteins were analyzed by either silver staining or Western blotting using streptavidin-HRP before mass spectrometry.

### Mass spectrometric analysis

Mass spectrometric analysis was carried out on a Thermo-Fisher Scientific LTQ Orbitrap Velos Pro mass spectrometer (Thermo-Fisher Scientific, Waltham, MA) interfaced with a Waters Acquity UPLC system (Waters, Milford, MA). The proteins bound to streptavidin beads (biotinylated proteins) and IgG beads (TgMyoA) were directly digested by trypsin. Samples were first reduced with 10 mM DTT in 10 mM ammonium bicarbonate and then alkylated with 55 mM iodoacetamide (prepared freshly in 10 mM ammonium bicarbonate). Alkylated samples were digested by trypsin (Promega, Madison, WI) overnight at 37°C. Tryptic peptides were first injected onto a C18 trapping column (NanoAcquity UPLC Trap column 180μm x 20mm, 5μm, Symmetry C18) and subsequently onto an analytical column (NanoAcquity UPLC column 100μm x 100mm, 1.7μm BEH130 C18). Peptides were eluted with a linear gradient from 3 to 40% acetonitrile in water with 0.1% formic acid developed over 90 minutes at room temperature at a flow rate of 500 nL/min, and the effluent was electro-sprayed into the LTQ Orbitrap mass spectrometer. Blanks were run prior to the sample to make sure there were no significant background signals from solvents or the columns. Database search against *Toxoplasma gondii* GT1 strain annotated proteins from ToxoDB (release 10.0, updated January 31, 2014) was performed using Sequest (Thermo-Fisher Scientific) search engine to identify biotinylated proteins and TgMyoA post-translational modification analysis was performed using the Thermo-Fisher Scientific Proteome Discoverer software (v2.0).

### Purification of recombinant TgCDPK3

The N-HIS-tagged TgCDPK3 expression construct described previously [14] was transformed into BL21-Rosetta (DE3)pLysS cells, which were then induced to express recombinant protein at 37°C with IPTG. His tagged recombinant protein was purified under native conditions using QIAexpress Ni-NTA fast start kit (Qiagen) according to manufacturer’s protocol. The kinase activity of recombinant TgCDPK3 was examined using peptide substrate syntide-2 (PLARTLSVAGLPGKK, AnaSpec, Inc.) and exhibited a specific activity of 22.9 μmol/min/mg.

### Peptide spot arrays

Peptide arrays were synthesized using SPOTs synthesis method and spotted onto a derivatized cellulose membrane (Intavis) as described previously [47]. The peptide membrane was blocked at room temperature for 30 minutes in binding buffer containing 5% BSA. Recombinant TgCDPK3 (5nM) was added to 50mM HEPES, pH 7.4, 100mM NaCl, 10mM MgCl2, 100μM ATP, 1mM CaCl2, 6μCi/ml [γ-32P]ATP and incubated at room temperature for 15 minutes. The membrane was washed three times with 100mM sodium phosphate pH 7.0, 1M NaCl, 10mM EDTA and visualized using phosphorimaging (Fuji phosphor imager). The phosphorylation of each peptide was detected and quantified using Multi Gauge version 3.0 (Fujifilm).

### Immunoblotting

Parasite lysates were heated at 100°C for 5 minutes in SDS-PAGE sample buffer with 2% 2-mercaptoethanol and resolved on 4–20% gradient gel (Bio-Rad, Hercules, CA). Proteins were transferred from the gel onto nylon membranes using a semidry transfer apparatus (Bio-Rad, Hercules, CA) at 12 V for 30 minutes. After blocking with 5% (w/v) skim milk powder in TBS, membranes were treated with rabbit anti-HA tag antibody (Cell Signaling Technology), for 1 hour. Membranes were then washed and incubated with horseradish peroxidase (HRP) conjugated goat-anti rabbit IgG (Sigma). After washing, membranes were treated with SuperSignal West Pico chemiluminescent substrate (Pierce Chemical) and imaged using FluorChem E (Proteinsimple) [43].

### Buffer switch assay and Phos-tag polyacrylamide gel electrophoresis

Intracellular parasites 24 hours post-infection were harvested in intracellular buffer [31], filtered with 3-μm Nucleopore membrane, pelleted and re-suspended in either intracellular buffer or extracellular buffer [31]. Parasites were then incubated at 37°C for 2 minutes and immediately placed on ice followed by centrifugation at 1000 g for 10 min at 4°C. The parasite pellet was then lysed with RIPA buffer containing phosphatase inhibitor, PhosSTOP (Roche) followed by addition of SDS sample buffer containing β-meracaptoethanol and heated at 100°C for 5 min. To examine phosphorylation status of Myosin-A, Phos-tag gel electrophoresis was carried out according to manufacturers instructions (Wako Chemicals, USA). Briefly 200 μM Phos-tag (Wako Chemicals, USA) and 100 μM MnCl_2_ were added to conventional 7.5% (w/v) acrylamide resolving gel and the gel was run at constant voltage at RT. The gel was washed three times in SDS-PAGE running buffer containing 10 mM EDTA and once each in running buffer and transfer buffer before transferring to a PVDF membrane for immunoblotting using anti-MyosinA antibody.

### Immunofluorescence microscopy

Immunofluorescence staining of intracellular parasites was performed according to previously described procedures [48]. The primary antibodies used were: mouse anti-HA (Cell Signaling Technology), and rabbit anti-GAP45 and rabbit anti-MLC1[23]. Secondary antibodies used include: Alexa Fluor-594- or Alexa Fluor-488-conjugated goat anti-rabbit or goat anti-mouse (Molecular Probes). Slides were viewed using a Nikon Eclipse E100080i microscope and digital images were captured with Hamamatsu C4742-95 charge-coupled device camera using NIS elements software.

### Ionophore induced egress assay

The efficiency of egress after calcium ionophore treatment was determined using established protocols [14]. Percent egress was determined by dividing the number of lysed vacuoles by the total number of vacuoles for a sample.

### Motility assay

Parasite motility assay was performed according to previously described methods [15, 23] with some modifications. Briefly, 24-well plates were pre-coated with 75 μg/ml of BSA in water at 37°C for 30 minutes and washed three times with intracellular buffer [31]. Intracellular parasites 24 hours post-infection were harvested in presence of intracellular buffer, filtered with 3-μm Nucleopore membrane, pelleted and re-suspended in intracellular buffer. The parasites were then added onto wells and allowed to settle for 20 minutes at 37°C and the plate was transferred onto a heated chamber (set at 37°C) of inverted microscope (Leica AF6000). The intracellular buffer in the well containing extracellular parasites was gently aspirated and extracellular buffer [31] was added. Forty seconds after exchanging the buffer, parasite motility was imaged for 2 minutes at 2 frames per second using LAS X software. The movies were then manually analyzed to determine parasites exhibiting either twirling or helical or circular gliding and the number of parasites performing each type of motility was normalized to the total number of parasites in each movie. The speed of the parasite gliding was determined by measuring the distance travelled in a given time by three motile parasites in each of two separate movies per strain. The experiments were repeated 3 times.

## Supporting Information

S1 Fig Mapping of TgCDPK3 phosphorylation sites on TgGAP45 by tiled peptide array analysis using purified recombinant TgCDPK3. Phosphorylation intensity of 15 amino acid length peptides that span full-length TgGAP45 and are each shifted by 3 amino acid was detected using MultiGauge version 3.0.
(TIF)

S2 Fig MS/MS fragmentation spectrum of Myosin A peptide containing Ser 743. The glideosome complex, which includes MyoA, was immunoprecipitated from intracellular parasites using an antibody against GAP45 and submitted to MS/MS. Spectrum of phosphorylated peptide sequence ^743^pSSKLPS*EEY*QLGKT*MVFLK^760^ of TgMyoA is shown. Asterisks indicate dehydrated serine, tyrosine, and threonine residues. The dominant neutral loss of phosphoric acid and water from the precursor ion and sequence specific fragment ions are labeled. The presence of y-series ions (y-6, y-11, y-14, and y-18) suggests that the first serine residue is phosphorylated. Detected fragment ions are shown in red (*b*-ions) and blue (*y*-ions).
(TIF)

S1 Table List of primers used in the study. All primers are in 5’ to 3’ orientation.
(DOCX)

S2 Table List of proteins that were commonly biotinylated in MBE1.1 and MBE1.1 + CDPK3-BirA* parasites. (DOCX)
