# Supplementary figures and images for: Phosphorylation of a Myosin Motor by TgCDPK3 Facilitates Rapid Initiation of Motility during Toxoplasma gondii egress
Source: PLoS Pathog. 2015 Nov 6;11(11):e1005268. doi: 10.1371/journal.ppat.1005268 (PMC4636360; doi:10.1371/journal.ppat.1005268)

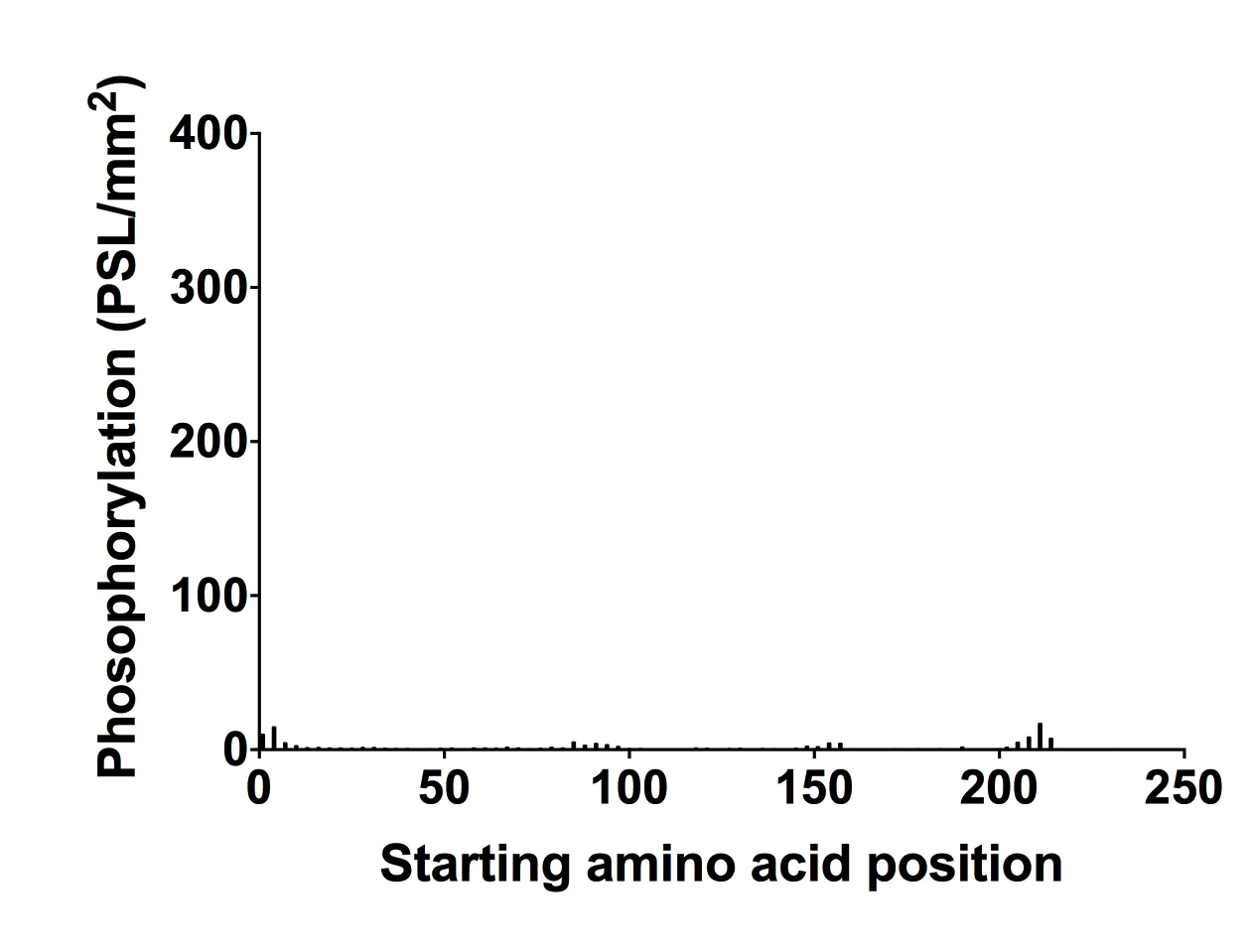

Supplement: S1 Fig — Phosphorylation intensity of 15 amino acid length peptides that span full-length TgGAP45 and are each shifted by 3 amino acid was detected using MultiGauge version 3.0. (TIF) [file ppat.1005268.s001.tif]

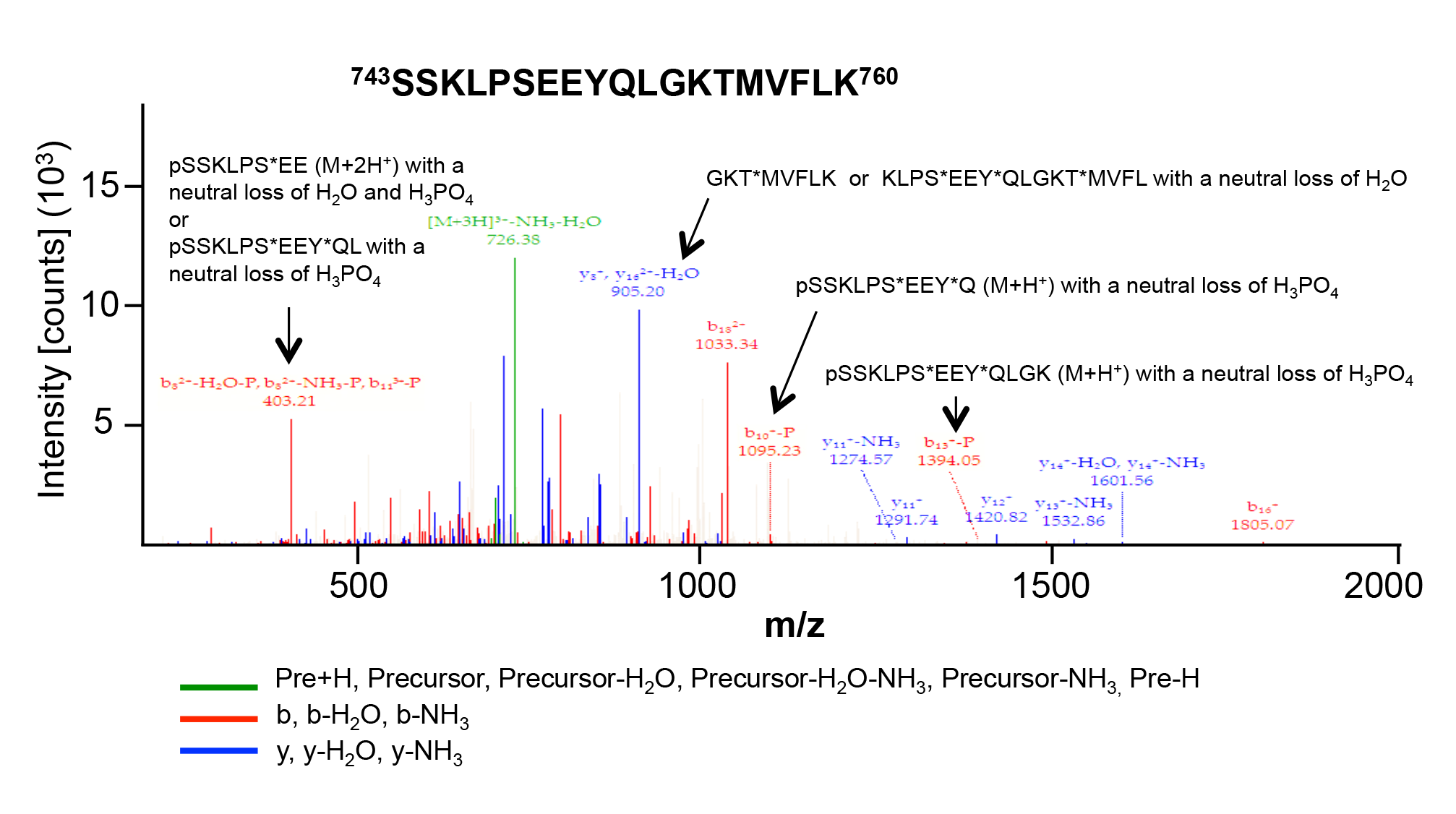

Supplement: S2 Fig — The glideosome complex, which includes MyoA, was immunoprecipitated from intracellular parasites using an antibody against GAP45 and submitted to MS/MS. Spectrum of phosphorylated peptide sequence 743pSSKLPS*EEY*QLGKT*MVFLK760 of TgMyoA is shown. Asterisks indicate dehydrated serine, tyrosine, and threonine residues. The dominant neutral loss of phosphoric acid and water from the precursor ion and sequence specific fragment ions are labeled. The presence of y-series ions (y-6, y-11, y-14, and y-18) suggests that the first serine residue is phosphorylated. Detected fragment ions are shown in red (b-ions) and blue (y-ions). (TIF) [file ppat.1005268.s002.tif]
