# Supplementary material for: Phosphorylation of a Myosin Motor by TgCDPK3 Facilitates Rapid Initiation of Motility during Toxoplasma gondii egress
Source: PLoS Pathog. 2015 Nov 6;11(11):e1005268. doi: 10.1371/journal.ppat.1005268 (PMC4636360; doi:10.1371/journal.ppat.1005268)
Supplement: S1 Table — All primers are in 5’ to 3’ orientation. (DOCX) [file ppat.1005268.s003.docx]

S1 Table

| **Primer Name** | **Sequence** |
| --- | --- |
| MyoA.S21A.FM | GCGCTGAAGAAGAGGTCGGCCGATGTCCACGCGGTC |
| MyoA.S21A.RM | GACCGCGTGGACATCGGCCGACCTCTTCTTCAGCGC |
| MyoAS743A.FM | CTGAGACTCCTCAAAGCTAGCAAACTGCCCAGC |
| MyoAS743A.RM | GCTGGGCAGTTTGCTAGCTTTGAGGAGTCTCAG |
| MyoA.20-21D.FM | GCGCTGAAGAAGAGGGATGACGATGTCCACGCGGTC |
| MyoA.20-21D.RM | GACCGCGTGGACATCGTCATCCCTCTTCTTCAGCGC |
| MyoA.743-744D.FM | CTGAGACTCCTCAAAGACGACAAACTGCCCAGCGAA |
| MyoA.743-744D.FM | TTCGCTGGGCAGTTTGTCGTCTTTGAGGAGTCTCAG |
