# Supplementary material for: Phosphorylation of a Myosin Motor by TgCDPK3 Facilitates Rapid Initiation of Motility during Toxoplasma gondii egress
Source: PLoS Pathog. 2015 Nov 6;11(11):e1005268. doi: 10.1371/journal.ppat.1005268 (PMC4636360; doi:10.1371/journal.ppat.1005268)
Supplement: S2 Table — (DOCX) [file ppat.1005268.s004.docx]

S2 Table

| **Gene ID** | **Annotation** |
| --- | --- |
| TGGT1_221230 | Acetyl-CoA carboxylase ACC1 |
| TGGT1_284190 | Pyruvate carboxylase |
| TGGT1_245460 | 40S Rbosomal protein S8, putative |
| TGGT1_294800A | Putative elongation factor 1-alpha (EF-1-ALPHA) |
| TGGT1_288720 | 60S Ribosomal protein L10, putative |
| TGGT1_263300 | Porin, putative |
